# Membrane mediated motor kinetics in microtubule gliding assays

**DOI:** 10.1038/s41598-019-45847-z

**Published:** 2019-07-03

**Authors:** Joseph Lopes, David A. Quint, Dail E. Chapman, Melissa Xu, Ajay Gopinathan, Linda S. Hirst

**Affiliations:** 1 Department of Physics, University of California, Merced, CA 95343 USA; 2 Center for Cellular and Biomolecular Machines (CCBM), University of California, Merced, CA 95343 USA; 3 Developmental and Cell Biology, University of California, Irvine, CA 92697 USA; 4 Department of Bioengineering, University of California, Merced, CA 95343 USA

**Keywords:** Biological physics, Membrane biophysics, Kinetics

## Abstract

Motor-based transport mechanisms are critical for a wide range of eukaryotic cell functions, including the transport of vesicle cargos over long distances. Our understanding of the factors that control and regulate motors when bound to a lipid substrate is however incomplete. We used microtubule gliding assays on a lipid bilayer substrate to investigate the role of membrane diffusion in kinesin-1 on/off binding kinetics and thereby transport velocity. Fluorescence imaging experiments demonstrate motor clustering on single microtubules due to membrane diffusion in the absence of ATP, followed by rapid ATP-induced dissociation during gliding. Our experimental data combined with analytical modeling show that the on/off binding kinetics of the motors are impacted by diffusion and, as a consequence, both the effective binding and unbinding rates for motors are much lower than the expected bare rates. Our results suggest that motor diffusion in the membrane can play a significant role in transport by impacting motor kinetics and can therefore function as a regulator of intracellular transport dynamics.

## Introduction

Within the cell, molecular motors play an important role in the transportation of intracellular cargo^1^ and contribute to the highly-organized nature of cellular function. By converting chemical energy into mechanical energy through ATP hydrolysis^2,3^ one of the main motor proteins, kinesin-1, carries cargo, typically contained within lipid vesicles^4,5^, along microtubule pathways. Such vesicles contain organelles and protein bodies enclosed within a fluid-like lipid bilayer, to which kinesin-1 is physically coupled either directly or through a protein linkage^6^.

Several different *in-vitro* approaches have been used previously to investigate motor-based transport. These primarily involved the transport of solid cargos such as silica beads or nanoparticles by motors along microtubules attached to glass^7–10^, or gliding assays on glass, where motors are deposited on the flat surface and microtubules, that are propelled by the stationary motors, are motion tracked^11,12^. The most striking difference between these *in-vitro* studies and the physiological system is the lack of the membrane. In the cell, cargos are coupled to motors via a lipid bilayer which is effectively a 2D fluid in which lipid molecules and included membrane proteins diffuse laterally. The substrate fluidity could result in significant differences in the coupling between motors attached to the same cargo, the forces experienced by them and even the binding and unbinding kinetics, leading to qualitatively different transport behavior. Despite this, very few studies of motor function using membrane-based cargos have been carried out to date and the impacts of membrane coupling on motor-based transport are only beginning to be explored^13–15^. For example, in a recent report, Grover *et al*.^15^ used gliding assays to demonstrate that kinesin-based transport velocity was negatively impacted by the presence of a lipid bilayer. Increases in motor-based transport velocity have been reported for membrane-bound myosin motors^13^ as well as kinesin motors^16^ and found to be sensitive to membrane composition.

In this work, we investigate a related question: the impact of fluid membrane substrates on kinesin-microtubule binding kinetics. In particular, we want to know if lateral membrane diffusion can significantly impact kinesin-microtubule on/off binding rates before and during transport. In the limit where motors do not crowd each other in the membrane, a mechanism to transport motors towards and away from the microtubule can alter the availability of motors for binding. Overall, the diffusion in the membrane along with the intrinsic on/off rate constants can set the time dependent density profile of motors in the vicinity of the microtubule. This in turn can potentially determine the number of engaged motors and hence transport characteristics such as processivity. An equivalent process can occur in the *in vivo* vesicle transport case, where motor density gradients can develop on the vesicle surface leading to clustering of motors near the microtubule and thus potentially enhanced transport.

To probe these questions, we carried out a series of gliding experiments based on the standard microtubule gliding assay^17,18^, but with the Kinesin-1 motors on lipid bilayer coated substrates as in^15^. Applying the membrane to a flat substrate allows us to take advantage of more convenient imaging possibilities and the use of a quasi-infinite bath of motors. The experimental gliding assay can be used to easily investigate transport over large distances^19^, as opposed to bead-cargo methods^20,21^ in which the motion of individual cargo-coupled beads is analyzed, but the surface of the bead cannot be imaged. Since biological kinesin cargos (e.g. lipid vesicles in the cell) are typically large compared to the nano-scale motors^22^, this flat geometry is a reasonably good experimental model to use. It should be noted however that in the cell, membrane vesicles are not rigid and that their local curvature may be impacted by motor binding and unbinding to the microtubule. These higher order effects are neglected in this paper but may be important for future investigations.

In this setup, Kinesin-1 molecules, which are directly coupled to the lipid bilayer, undergo Brownian diffusion, the magnitude of which is dependent on the effective hydrodynamic radius of the motor and the composition of the membrane. To quantify the effect of the lipid bilayer on kinesin binding to microtubules, we designed an experiment in which the contribution of membrane diffusion to the effective on-rate (${k}_{a}^{eff}$) could be assessed. We measured the fluorescence intensity due to labeled kinesin binding on single microtubules. In the absence of ATP we observed an increase in kinesin fluorescence intensity on the stationary microtubules purely due to the irreversible binding of motors that arrived at the microtubule via diffusion in the membrane. The introduction of ATP led to a rapid exponential decrease in kinesin fluorescence intensity as the motors begin to walk along the microtubule and disassociate stochastically during the stepping process. An analytical model was used to interpret the data and extract effective binding and unbinding rates. In addition, we altered the kinetics by changing ATP concentration and measured ATP-dependent steady-state gliding velocities on the membrane. Our experimental data combined with analytical modeling show that the diffusion controlled on/off binding kinetics of the motors are much slower than the expected bare rates. Our findings underline the importance of including membrane diffusion in models of motor kinetics, an aspect of kinesin transport that is only beginning to be explored.

## Results

### Direct observation of motor clustering on microtubules due to membrane diffusion

**Figure 1:**
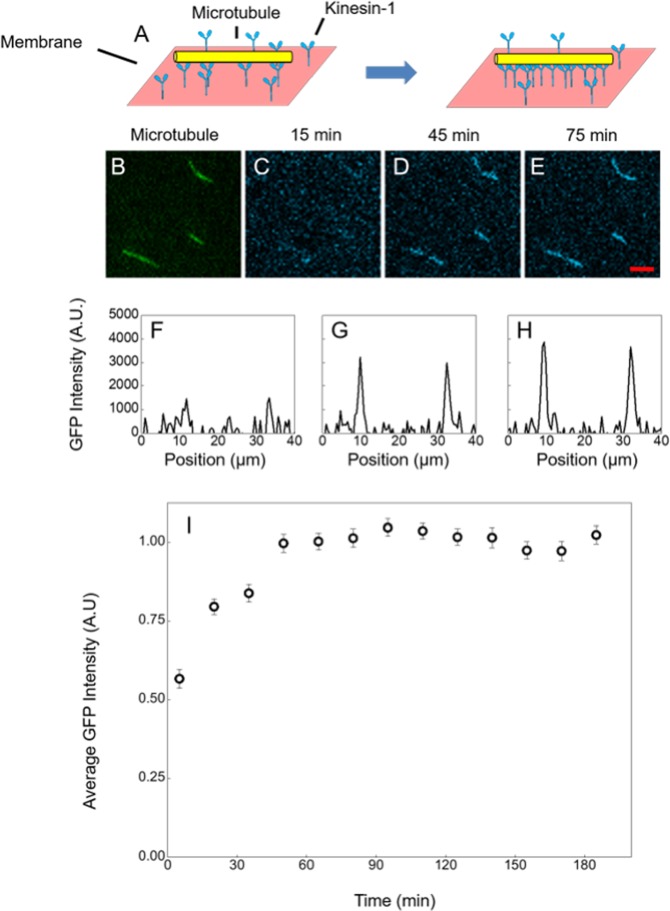
Membrane-coupled motors accumulate on static microtubule via membrane diffusion. (**A**) Cartoon schematic of motor protein clustering on a microtubule. (**B–E**) Fluorescence images of microtubule and GFP labeled kinesin clustering on an immobile microtubule recorded at 15, 45 and 75 minutes and (**F–H**) corresponding 1D line profiles of image intensity after background subtraction, corresponding to peaks of intensity. (**I**) Normalized GFP intensity as a function of time calculated from the average area under the peak for 45 microtubules. Error bars are calculated from the standard error of the mean, scale bar is 5 µm.

To quantify the effect of membrane diffusion on Kinesin-1 binding, fluorescence microscopy experiments were performed. Since motor proteins are expected to bind and remain attached to the microtubule in the absence of ATP, we were able to observe the membrane coupled GFP-labeled kinesin motors build up as they diffuse into the regions surrounding the microtubules. Figure 1 shows (Fig. 1A) a schematic of the process and microscope images of the Rhodamine-labeled microtubules (Fig. 1B) and three images of GFP-labelled kinesin recorded at 15 min intervals (Fig. 1C–E). These images clearly illustrate the build-up. To quantify the average increase in intensity we performed 1D cross plots of the microtubules (Fig. 1F–H show corresponding plots to images Fig. 1C–E). We calculated the area under the curves for each microtubule and averaged the values across 45 microtubules for each observation time to obtain (Fig. 1I) GFP intensity as a function of time. This graph shows the average intensity build-up reaching saturation within 1 hr.

**Figure 2:**
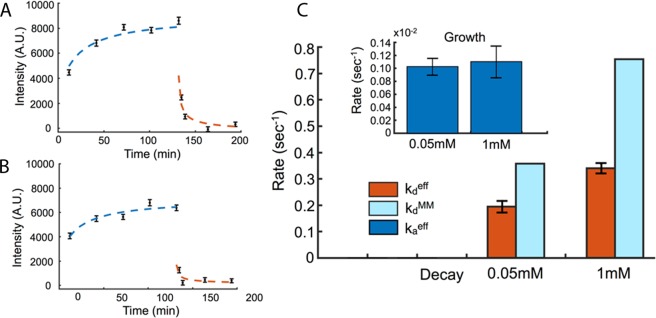
Membrane-coupled motors disassociate rapidly with the introduction of ATP. Experimental measurements of GFP intensity as a function of time for 45 microtubules using the methods described in Fig. 1. Data shown for (**A)** 0.05 mM and (**B**) 1 mM ATP, where ATP is introduced to induce gliding at 120 mins. Error bars indicate standard error of the mean. Theoretical fits (dashed lines) are performed separately for kinesin aggregation without ATP (Blue) and the decay of the microtubule-bound kinesin signal after ATP addition (Orange). (**C**) Rates ${k}_{a}^{eff}$ (inset) and ${k}_{d}^{eff}$ extracted from our fitting model (Eqs 3b and 5b) for ATP concentrations 0.05 mM and 1 mM (Orange) show a general trend of increasing off rate (${k}_{d}^{eff}$) with increasing ATP concentration. Both growth rates (${k}_{a}^{eff}$) are approximately the same (dark blue). The fitted effective off-rates are compared with expected ${k}_{d}^{MM}\,$values calculated assuming Michaelis-Menten kinetics (light blue).

Having verified the effects of membrane diffusion on kinesin binding in the absence of ATP, we then performed a second experiment in which ATP was introduced into the system. Figure 2A, B show GFP intensity (measured in the same way as for Fig. 1) as a function of time before and after ATP introduction. ATP was introduced to the flow cell at two different concentrations (1 mM and 0.05 mM) after which time we observed a steep decay in GFP intensity and the onset of gliding motion, consistent with rapid kinesin unbinding.

To further analyze the experimental data presented in Fig. 2A and compare the curves before and after ATP addition we constructed an analytical model for kinesin binding and unbinding onto the microtubules. In the absence of ATP, microtubules remain static in space and as time passes the intensity of GFP-labeled kinesin grows. After the addition of ATP, the kinesin signal drops dramatically at an extraordinary rate. We see that the two intrinsic rates that govern this process are very different, but also slow compared to the intrinsic motor on and off rates which are on the inverse second scale, suggesting that both rates may be influenced by a diffusion limited process.

Assuming the fluorescence intensity of kinesin in our images is directly proportional to the number density of kinesins on the microtubule, we can directly measure this density as a function of time. The dynamics governing the number density of kinesin per unit length, *σ* (*t*), on the microtubule are coupled to the number density of kinesin per unit area, *c*(*x*, *t*), bound to the membrane and is given by^23^,

1a $$\frac{d\sigma }{dt}={k}_{a}c(0,t)({\sigma }_{m}-\sigma )-{k}_{d}\sigma $$

1b $$\frac{d\sigma }{dt}=D{\frac{d}{dx}[c(x,t)]|}_{x=0}$$

where *x* is the distance away from a microtubule in the direction perpendicular to its axis, *k*_*a*_ and *k*_*d*_ are the intrinsic kinesin association rate and disassociation at the microtubule surface respectively and *D* is the diffusion constant of the lipid bound kinesins on the substrate. Equation 1a therefore describes the binding kinetics from the available membrane-bound kinesin motor concentration at the microtubule surface (*c* (0, *t*)), where *σ*_m_ is the maximum/saturated motor coverage that is controlled by the available binding sites on the microtubule. The flux at the interface between the surface bound kinesin and the microtubule must also satisy Eq. 1b. The membrane bound kinesin concentration, *c*(*x*, *t*), is governed by a 1D diffusion equation with diffusion constant *D*, initial condition *c* (*x*, *0*) = *C*_0_ and boundary conditions set by Eq. 1b, c (∞, *t*) = *C*_0_, where *C*_0_ is the ‘bulk’ membrane bound concentration of motors far away from the microtubules^23^. Note that we assume translational invariance parallel to the microtubule, i.e. we ignore edge effects, which seems reasonable given the fairly uniform coverage along the length. The dissociation rate of kinesin, *k*_*d*_, from the microtubule surface is ATP-dependent and hence can be turned off by removing ATP from the system.

### No ATP case

In the absence of ATP (*k*_*d*_ = 0), Eq. 1 can be solved, along with the coupled equation and constraints on *c*(*x*, *t*), to find the microtubule coverage *σ* (*t*) in the diffusion controlled regime (see^23^ for details) which is given by

2 $$\sigma (t)={\sigma }_{m}(1-exp(\frac{-2{C}_{0}}{{\sigma }_{m}}\sqrt{\frac{Dt}{\pi }}))$$

which in turn can be expressed as

3a $$\sigma (t)={\sigma }_{m}(1-exp(-\sqrt{{k}_{a}^{eff}t}))$$

3b $${k}_{a}^{eff}=\frac{4D{C}_{0}^{2}}{{\sigma }_{m}^{2}\pi }$$

Fitting the observed increase in kinesin at the microtubule provides an estimate for the effective association rate, *k*_*a*_^*eff*^ as defined in Eq. 3a,b (Fig. 2C – inset). We find that the effective association rate is two orders of magnitude smaller than the experimentally determined rates for single kinesin/microtubule systems where the kinesin is in close proximity to the microtubule, indicating the effects of both dilution and diffusion^24^. It is also important to note that the saturation is controlled by an exponential that depends on the square root of *t* rather than *t*, which is also qualitatively slower. This result shows that diffusion in the lipid bilayer can significantly alter kinesin-microtubule association kinetics.

From the fit of Eq. 3a we may also estimate the surface DGS-NTA bound kinesin concentration, *C*_0_, in our system from the effective association rate (Eq. 3b). To do this, we need to estimate the other parameters controlling *k*_*a*_^*eff*^. We can estimate the saturated coverage, *σ*_m_, by considering that kinesin, when attached to the microtubule, will occupy a distance along a single protofilament of 32 nm. Furthermore, we assume that motors from the membrane can reach at most three protofilaments on the microtuble nearest the membrane surface, given that their stalk rest length is about 40 nm. Assuming that each kinesin occupies two neighboring beta subunits along the same protofilament and neighboring kinesin do not share beta subunits we calculate the coverage at saturation to be *σ*_m_ = 1000 nm/µm × 3/32 nm ≈ 93.8 µm^−1^.

Using these estimates, we find that the number density of the kinesin bound to DGS-NTA, *C*_0_ = 25.4 ± 0.62 µm^−2^, which is a bit larger than those used in previous studies^15^ and is reasonable because we chose to use a higher concetration of motors.

Finally, we note that the process is diffusion controlled when the control parameter *γ* (Eq. 4) is small.

4 $$\gamma =\frac{D{C}_{0}}{{k}_{a}{\sigma }_{m}^{2}}$$

Using the values quoted above for *D*, *C*_0_ and *σ*_m_ and estimating *k*_*a*_ ~ 0.47 ± 0.01 µm^2^ s^−1^ from the early time slope of the numeric fit for *σ* (*t*), (fit to Eq. 3a and Fig. 2A,B), we find that *γ* ~ 6.8e-5 signifying that we are well within the diffusion controlled regime *γ* ≪ *1*.

### ATP case

When the system becomes active by the addition of ATP in general we have two more kinetic time constants at play. As motors begin to walk along a microtubule at each step they have a chance to disassociate or walk off the end of the microtubule. Since the microtubule lengths (~20 µm) here are significantly longer than the typical motor run lengths (800 nm), the effective off rate would be expected to just depend on the intrinsic kinesin disassociation rate *k*_*d*_. However, as we have seen in the adsorption process (previous section), the kinetics are diffusion controlled and we may expect a similar effect on the disassociation from the microtubule.

Equation 1 can also be exactly solved in this regime (*k*_*d*_ > 0, *γ* ≪ 1), and in the long time limit, the coverage is given by

5a $$\sigma (t) \sim {\sigma }_{m}\frac{{k}_{a}{C}_{0}}{{k}_{d}}(1-\frac{1}{\sqrt{{k}_{d}^{eff}t}})$$

5b $${k}_{d}^{eff}=\frac{D}{{\sigma }_{m}^{2}{k}_{a}^{2}}{k}_{d}^{2}$$

Again the diffusion controlled approach to steady state has a very different functional form from a normal exponential. Fitting the data (Fig. 2A,B) after the addition of ATP (*t* > 120 min) using Eq. 5a, we obtain the value for the effective off-rate, *k*^*eff*^_*d*_ (Fig. 2C). It is to be noted that, by assuming translational invariance, we are also implicitly ignoring the effect of the motion of the microtubule in this calculation. This is reasonable if the timescale set by the ratio of the microtubule length to the velocity (~60 s) is large compared to the timescale of the effective off-rate (~3–5 s).

Comparing the effective disassociation rate with the intrinsic rate expected from Michaelis-Menten at both ATP concentrations we see that they are very different, as indicated by Eq. 5b. The effective disassociation constant depends not only on the intrinsic disassociation rate but also on the diffusion constant, the association constant and the saturating motor concentration on the microtubule. This difference comes from the effect of diffusion of motors in the bilayer. Physically, this occurs because motors that disassociate from a microtubule persist in the area around the microtubule because diffusion is slow in the bilayer, giving them a chance to rebind. This gives rise to an effectively lower disassociation rate of motors from the microtubule surface, which depends on the binding rate, saturating concentration and also the diffusion constant. Thus the diffusion controlled process causes the fluorescence signal to remain higher than it should be as compared to kinetics where motors disassociate and are quickly assimilated into the bulk concentration.

### Effects of kinetics on gliding velocity

**Figure 3:**
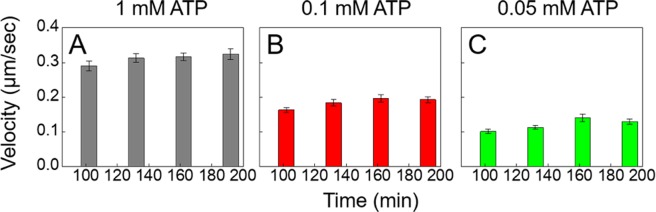
Membrane-coupled motors glide at lower speeds with lower ATP concentrations. Gliding experiments were carried out at 3 different ATP concentrations, 1 mM (**A**), 0.1 mM (**B**), and 0.05 mM (**C**). Microtubule gliding velocities were recorded at steady state after 2 hours. Error bars represent the standard error of the mean. Estimates for gliding velocities at 1 mM and 0.05 mM ATP are V_MT_(1 mM) ≈ 299.4 nms^−1^, V_MT_(0.1 mM) ≈ 196.3 nms^−1^ and V_MT_(0.05 mM) ≈ 141.9 nms^−1^.

To quantify the effects of perturbing kinetics on membrane-based gliding in our system we looked at the effect of ATP on steady state gliding velocity for three different ATP concentrations, 1 mM, 0.1 mM, and 0.05 mM. The data is presented in Fig. 3. We observed no time dependence on gliding velocities, and a trend that gliding velocity increases in magnitude as available ATP concentration in the system also increases. It is to be noted that ATP concentration is non-trivially coupled to the gliding velocity. Increasing the ATP concentration has two effects – it increases the intrinsic disassociation rate thereby reducing the steady state coverage of motors but also increases the speed of the individual motors. The gliding velocity is however directly proportional to the motor stepping speed but only weakly dependent on the steady state coverage at higher coverages^9^. Thus, as expected, the gliding velocity rises with ATP concentration consistent with Michaelis-Menten kinetics. We compared these steady state velocity values with the expected values obtained using our estimates for the steady state motor coverage *σ*(*t* → *∞*) ≈ *σ*_*m*_(*C*_0_ *k*_*a*_*/k*_*d*_) (Eq. 5a) and theoretical transport efficiencies given in [Grover *et al*.^9^]. We found very good agreement with our experimental gliding velocities (Fig. 3A,C) for both 1 mM and 0.05 mM ATP concentrations, V_MT_ (1 mM) ≈ 299.4 nms^−1^, V_MT_ (0.1 mM) ≈ 196.3 nms^−1^ and V_MT_ (0.05 mM) ≈ 141.9 nms^−1^.

## Discussion

In this study, our goal was to investigate if motor coupling to a fluid-like lipid membrane impacts kinesin-based transport through in-plane motor diffusion. In their recent study, Grover *et al*.^15^ investigated how kinesin-1 transport efficiency was impacted when bound to a lipid bilayer. Using gliding assays, they found that membrane-anchored motors exhibited reduced transport speeds at steady state and attributed the effect to motor slippage in the lipid bilayer. They also found that the steady state microtubule gliding velocity increased with increasing membrane-bound motor density. Our work focuses on the kinetics of the process and the approach to steady state. Our results reveal that transport via membrane-bound motors is impacted by additional kinetic mechanisms - the effects of diffusion on motor binding and unbinding rates and the effects of ATP on the unbinding rate influencing the steady state motor coverage. In a full model of motor transport, it will therefore be important to take the effects of motor diffusion into account.

We first examined kinesin binding rates in the absence of ATP to highlight the potential contribution of diffusion on transport. Without ATP on a non-diffusive substrate, the binding on-rate *k*_*a*_ should be zero, but in the case where kinesin is coupled to a fluid bilayer, motors can approach the static microtubule via diffusion, bind and build up on the microtubule while stationary, producing a diffusion controlled on-rate. Fluorescence microscopy allows us to observe this process clearly and plot kinesin accumulation on individual microtubules as time-dependent fluorescence intensity to a saturation point. Our theoretical modelling showed that this process is governed by diffusion and that the approach to saturation was exponential in *t*^*1/2*^ with an effective on rate ${k}_{a}^{eff}$ of approximately 0.1 min^−1^, which is an order of magnitude lower than what one would expect from a purely reaction controlled on-rate. It is interesting to note that the effective on rate in this diffusion controlled limit is independent of the intrinsic on rate but directly proportional to the diffusion constant (as in Eq. 3b), allowing the membrane fluidity to directly tune this on rate.

We next considered the effects of ATP. Kinesin off rates (*k*_*d*_) are dependent on ATP concentration and our fitted results for the effective off rates were compared to purely theoretical values of *k*_*d*_ as determined by Michaelis-Menten Kinetics using the steady state maximum velocities obtained in Fig. 3. We noticed that a simple Michaelis Menten model produced higher *k*_*d*_ values than those obtained from the fit to our analytical model. As indicated by Eq. 5b, the effective disassociation constant depends not only on the intrinsic disassociation rate but also on the diffusion constant, the association constant and the saturating motor concentration on the microtubule. This effect may be understood as occurring due to rapid rebinding of recently unbound motors surrounding the microtubule. Such an effect in vesicle transport where motors are coupled to a fluid membrane, could act to increase the number of active microtubule-bound motors and thus impact long-range transport in the cell.

It is interesting to note that the steady state coverage of motors (Eq. 5a at infinite t) does not depend on the diffusion constant. Thus while diffusion affects the effective on and off rates and hence the timescale of the transient approach to steady state, it does not directly affect the steady state coverage for microtubules in a bath of motors. However, in the case of a vesicle being transported along a microtubule, the presence of diffusion makes a qualitative difference in allowing all the motors on the surface to become available for binding as opposed to the case of a rigid bead where only the motors within geometrical reach are available. Thus the presence of diffusion breaks a common assumption about the availability of motors and significantly increases it. Transient time scales are also important in the vesicle case, because the time it takes for a second motor to bind the microtubule before the first motor disassociates is relevant for the probability of cargo disassociation. Finally, we note that ATP can change the intrinsic off-rate which in turn changes the steady state concentration as well. Thus, for example, reducing ATP decreases *k*_*d*_ while also increasing steady state motor coverage – leading to more motors that could potentially lead to increases in run length.

In real biological vesicles the presence of ordered lipid domains may additionally impact this process. For example, domains rich in lipids with saturated chains and cholesterol can exhibit lateral diffusion constants up to 10 times lower than those in the fluid lipid phase. It will be very interesting in future to consider the possible impact of membrane domains on kinesin-based transport.

The results from this study demonstrate that motor kinetics are an important determinant of transport that can be affected by membrane diffusion. Thus more accurate models and experimental systems which incorporate the lipid bilayer are needed to increase our understanding of motor-based transport.

## Methods

### Lipids and reagents

All lipids using in this work were purchased from Avanti Polar Lipids (Alabaster, AL, USA) in chloroform and used without further purification. They include 1,2-dioleoyl-sn-glycero-3-phophocholine (DOPC), 1,2-dioleoyl-sn-3-phosphoethanolamine-N-(7-nitro-2-1,3-benzoxadiazol-4-yl) (ammonium salt) (DOPE-NBD), and 1,2-dioleoyl-sn-glycero-3-[(N-(5-amino-1-carboxypentyl) iminodiacetic acid) succinyl] (ammonium salt) DGS-NTA. DGS-NTA was selected as a lipid to anchor motor proteins due to its similar alkyl chain structure to DOPC and ability to coexist in a liquid disordered phase.

### Microtubule and kinesin motor preparation

Porcine tubulin (Cat. T240), and rhodamine labeled porcine tubulin (Cat. TL590M) were purchased from Cytoskeleton, Inc (Denver, CO, USA). Labeled and unlabeled porcine tubulin was dissolved at a ratio of 1:5 at a concentration of 1.5 mg/ml in PEM80 (80 mM PIPES, 1 mM ethylene glycol bis (β-aminoethyl ether), 1 mM MgSO_4_, pH 6.9), buffer and supplemented with 10 mM GTP and 1 mM taxol). The tubulin solution was then incubated in a 37 °C bath for 12 hours to allow for polymerization after which microtubules are then stored at room temperature in a dark box. Recombinant penta-histidine-tagged kinesin protein was purified from e coli as previously described^19,20^. All other chemicals were purchased from Sigma Aldrich (St. Louis, MO, USA).

### Lipid membrane preparation

Lipid mixtures of 89.95 mol% DOPC, 10 mol% DGS-NTA, and 0.05 mol% DPPE-NBD (fluorescent lipid), were mixed in chloroform then vacuum dried to remove all chloroform. Other lipid mixtures used included 80 mol% DOPC with 20 mol% DGS-NTA and 90 mol% DOPC with 10 mol% DGS-NTA. Lipids were then rehydrated with nanopore water to final concentration. To form bilayers, we used the vesicle fusion method: small unilamellar vesicles (SUVs) were generated via tip sonication^24^ and drop-cast onto an open clean glass flow cell coated with BSA. The substrate was incubated at 50 °C for 1 hour to allow for vesicle fusion on the surface forming a complete single bilayer. Bilayers were verified using fluorescence microscopy before sealing the flow cells.

To recruit kinesin motors onto the membrane, we included the lipid 1,2-dioleoyl-sn-glycero-3-[(N-(5-amino-1-carboxypentyl)iminodiacetic acid) succinyl] (ammonium salt) (DGS-NTA) at 10 mol%, unless otherwise specified, in a bilayer comprised primarily of 1,2-dioleoyl-sn-glycero-3-phophocholine (DOPC). The membrane was designed to exhibit homogeneous fluid behavior while incorporating triple carboxyl group binding sites to provide nonspecific binding through electrostatic attraction and hydrogen bonding to the kinesin motor’s histidine tag^25–27^. *In-vivo*, motors are known to bind to vesicles by a variety of different mechanisms that involve specific interactions with a complex set of proteins that assemble on the membrane cargo. The architecture of these assemblies is, in general, poorly understood^28^. For our experiments the aim was not to accurately reproduce an *in-vivo* binding mechanism, but to create a simple linkage that would introduce quantifiable motor diffusion to the system.

Motor-lipid binding was quantified using confocal fluorescence microscopy (Supplemental Fig. S2). The lipid membranes were decoupled from the underlying glass substrate by a bovine serum albumin (BSA) cushion, to minimize any possible interactions with the glass surface that might influence membrane fluidity. The bilayers are constructed inside a flow cell such that kinesin motors are introduced to bind to the available DGS-NTA binding sites. Microtubules are then flowed across the membrane and bind to the tethered motors.

### Flow cell preparation

Flow cells^19^ for the experiments are constructed by placing two thin strips of tape on a glass slide. Glass was sonicated in Acetone, Methanol, Ethanol, and Nanopure water for 1 hour each and dried with N_2_ gas. After forming the flowcell, 5 mg/ml of BSA was dropcast onto the slide and incubated at 50 °C for 2 hours. Excess BSA was removed with 1 ml of nanopure water.

After deposition of the lipid bilayer, a glass coverslip is placed on top of the tape and set in place with wax to form the flow cell with a volume of approximately 15 μl. Reagents are added into the flow cell, which is then sealed with vacuum grease to prevent drying.

### Microtubule gliding experiments

Kinesin was prepared using a previously reported method^20^. Kinesin solutions, 300 nM in PEM80 buffer, were introduced into the flow cell and incubated for 10 min. After allowing 10 minutes for the GFP labeled motors to adhere to the surface, we flowed in the 1:5 rhodamine labelled Microtubules diluted in PEM80 (10 µM Taxol), which also removed excess motors. Microtubules were then given 10 minutes to adhere to the remaining motors bound on surface. Lastly a motility mix (PEM80 supplemented with 1 mM ATP, 1 mM DTT, 10 uM taxol, 0.22 mg/ml glucose oxidase, 0.04 mg/ml catalase, 3.68 mg/ml glucose, 2 mM phosphocreatine and 70 μg/mL creatine phosphokinase)^19^ was added to provide a regenerative ATP source and reduce bleaching.

### Motor clustering experiments

To measure motor protein binding onto the static microtubules, Green Fluorescent Protein (GFP) tagged kinesin-1 motor proteins were used and GFP intensity adopted as a proxy for number of proteins. Microtubules were deposited onto a lipid membrane with motor proteins as in the above experiments. Excess unbound microtubules were removed from the flow cell with a wash buffer (PEM80, 1 mM DTT, 10 uM Taxol, 0.22 mg/ml glucose oxidase, 0.04 mg/ml catalase, and 3.68 mg/ml glucose) and the chamber sealed with vacuum grease. Images were recorded on a fluorescence microscope every 15 minutes for three hours then a final image was recorded of Rhodamine-labelled microtubules to confirm their physical location.

### Imaging and data analysis

Gliding experiments were imaged using a Leica Microsystems Inc. DM 2500 P fluorescence Microscope, (Buffalo Grove, IL, USA) and a QImaging Retigia Exi camera (Surrey, BC, Canada). In a typical experiment Tif images are collected at 4–8 observation areas per slide, to produce gliding movies for 1.5 min per area using a 20x or 63x objective. Images were recorded at 10 second intervals with a 500 μs exposure time and 0.69 s delay between frames. All image analysis was carried out using Image Processing and Analysis in Java (ImageJ, http://imagej.nih.gov/ij/). Microtubule gliding velocities were tracked using the MTrackJ plugin for ImageJ (http://www.imagescience.org/meijering/software/mtrackj/). Microtubules where tracked by their leading edge, with an average of 20 microtubules tracked per analysis region. For confocal imaging of the lipid membrane (Supplemental Fig. 1), a Zeiss LSM 880 with AiryScan + FAST was used, and subsequent analysis carried out using bio-formats in Image j (https://docs.openmicroscopy.org/bio-formats/5.7.0/users/imagej/).

### Fluoresence recovery after photobleaching (FRAP)

Membranes were prepared in identical fashion to those used for gliding experiments. Kinesin-1 at a concentration of 328 nM was introduced into the flowcell and left to bind for 10 minutes. Afterwards 600 ul of PEM80 supplemented with 2 mM DTT was flowed in to remove unbound protein from the chamber. The labeled protein was imaged using the same fluorescent microscope using a 63x water immersion objective, and subsequently bleached by overexposing the region for 10 minutes. The bleached region was imaged every 10 minutes for two hours and diffusion constants calculated using a well established method^26^.

## Supplementary information

Supplementary Information

## Data Availability

The datasets generated during and/or analysed during the current study are available from the corresponding author on reasonable request.
