# Supplementary material for: Membrane mediated motor kinetics in microtubule gliding assays
Source: Sci Rep. 2019 Jul 3;9:9584. doi: 10.1038/s41598-019-45847-z (PMC6610617; doi:10.1038/s41598-019-45847-z)
Supplement: Supplementary file 1 — Supplementary Information [file 41598_2019_45847_MOESM1_ESM.docx]

**Membrane mediated motor kinetics in microtubule gliding assays**

Joseph Lopes^1^, David A. Quint^1,2^, Dail E. Chapman^3^, Melissa Xu^4^, Ajay Gopinathan^1,2^ and Linda S. Hirst^1^

^1^Department of Physics, University of California, Merced, CA 95343, USA

^2^Center for Cellular and Biomolecular Machines (CCBM), University of California, Merced, CA 95343, USA

^3^Developmental and Cell Biology, University of California, Irvine, CA 92697, USA

^4^Department of Bioengineering, University of California, Merced, CA 95343, USA

SUPPLEMENTARY INFORMATION

**Supplementary Figure S1**

**Supplementary Figure S2**

**Supplementary methods.**


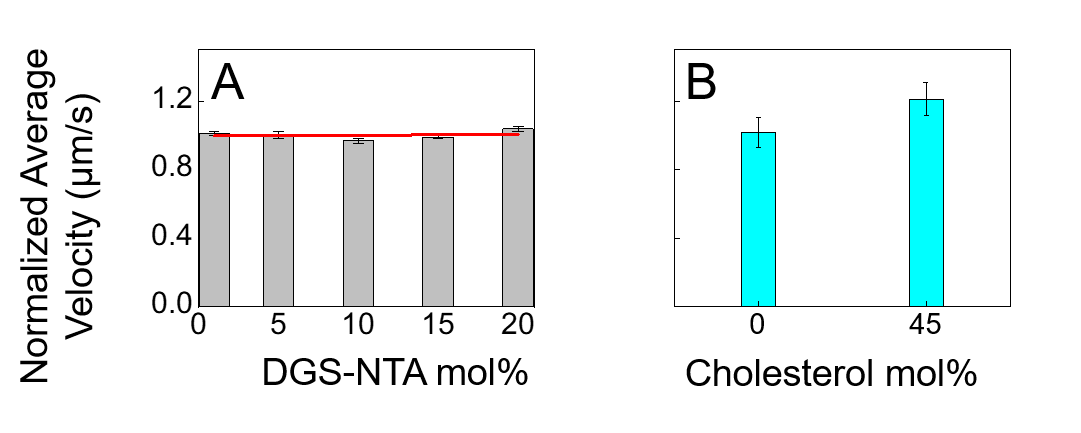


**FIGURE S1 Membrane-coupled motor gliding velocity is independent of DGS-NTA concentration.** Gliding experiments were carried out with (A) membranes composed of DOPC and 1-20 mol % DGS-NTA lipid and (B) at 0 and 45 mol % cholesterol. Velocities were recorded for percentage and normalized by the average gliding velocity of all populations. The gliding velocity shows no significant change relative to DGS-NTA content, but is impacted by the addition of cholesterol. Error bars represent the standard error of the mean.

To look at the dependence of microtubule gliding on membrane motor density, we measured gliding velocities as a function of membrane composition, in which we varied the density of DGS-NTA lipids. Results presented in Figure S1 indicate no systematic dependence on DGS-NTA membrane density (and therefore membrane-bound kinesin concentration) within the range of 5-20% DGS-NTA lipids. Our experiment was designed to provide a plentiful supply of motors to the microtubules and thus is relatively insensitive to binding site concentration.

We noted also that modifications to membrane composition can impact gliding velocity for our system. We prepared membranes to include 45mol % cholesterol and as predicted in (9) the gliding velocity was reduced by approximately 30%.


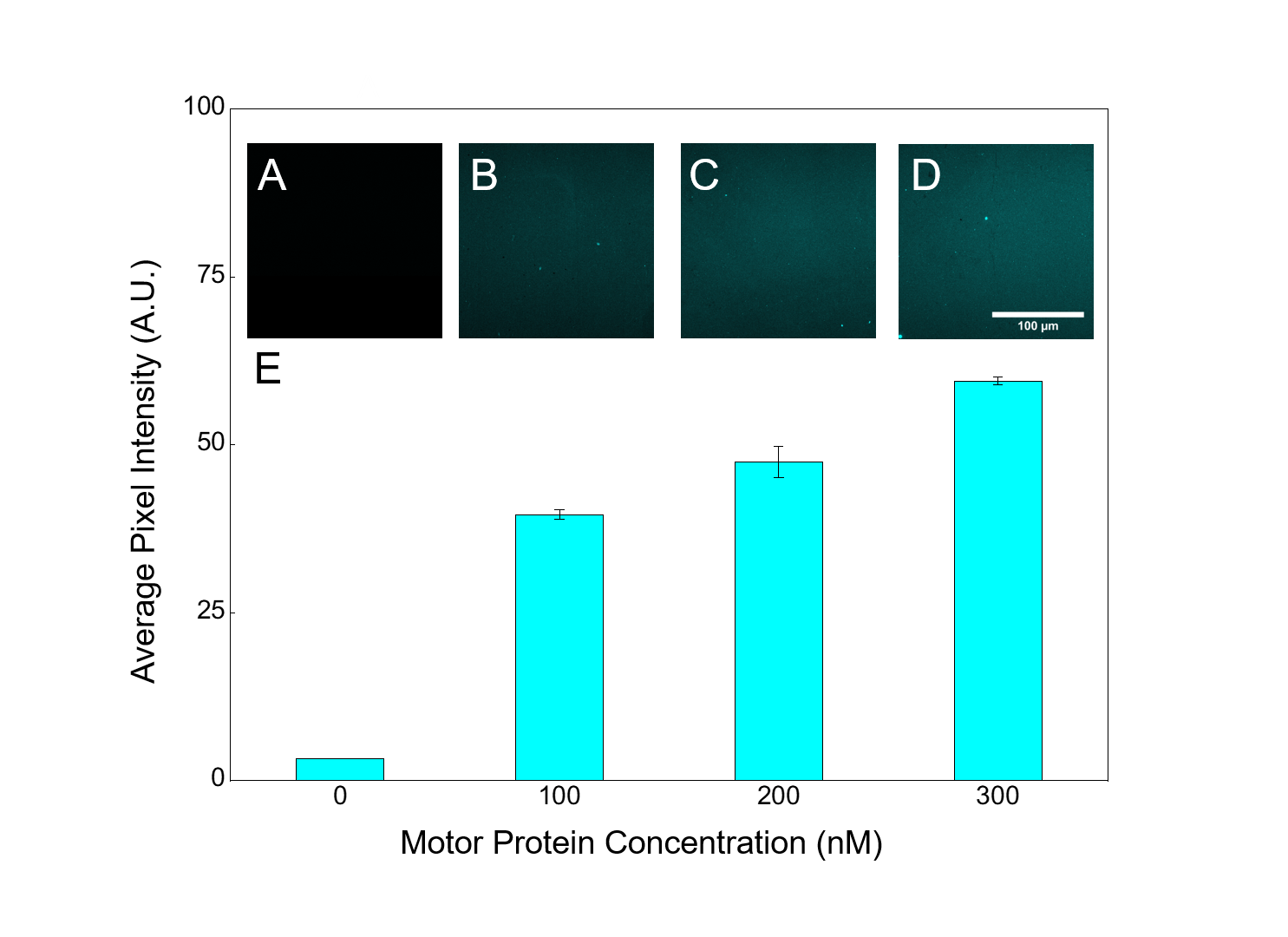


**FIGURE S2. Kinesin Non-Specifically Binds to DGS-NTA lipids.** Confocal images of the lipid membranes composed of DOPC and varying concentrations of DGS-NTA after introduction of GFP labeled kinesin-1, whose concentration in solution was 0 nM (*A*), 100 nM (*B*), 200 nM (*C*), and 300 nM (*D*) to quantify non-specific binding. Scale bar represents 50 µm. (*E*) Average intensity of GFP signal plotted against motor protein concentration, with error bars representing the standard error of the mean (SEM). This plot demonstrates the increased motor concentration on the surface with the increased presence of motor proteins in solution.

**Supplementary method. Motor Proteins Non-Specifically bind to NTA group.**

GFP labeled kinesin-1 motor proteins were added to a flow cell in a buffer exchange process. These motor proteins would adhere to the lipid membrane non-specifically, attaching to the NTA group of the lipid molecule. This non-specific binding was assessed using confocal microscopy (Fig. S1), to demonstrate that increased motor protein concentration in buffer would translate increased concentration on the surface. Qualitatively we can see that motor proteins bind and remain uniformly distributed on a lipid membrane (Fig. S1 A - D). Buffers containing motor protein at different concentrations was added to the flow cell, and the buffer subsequently exchanged after 10 minutes to remove excess motors. By using the GFP signal of the motor protein as a proxy for concentration, we see that increased motor concentration on the surface after buffer exchange is a result of increased concentration in solution (Fig. S1 E).
